# The Aminoacyl-tRNA Synthetase and tRNA Expression Levels Are Deregulated in Cancer and Correlate Independently with Patient Survival

**DOI:** 10.3390/cimb44070207

**Published:** 2022-07-02

**Authors:** Anmolpreet Kaur Sangha, Theodoros Kantidakis

**Affiliations:** Aston Medical School, College of Health and Life Sciences, Aston University, Birmingham B4 7ET, UK; anmolpreetsangha@hotmail.co.uk

**Keywords:** aminoacyl-tRNA synthetase, ARS, tRNA, expression, cancer, TCGA, patient survival

## Abstract

Aminoacyl-tRNA synthetases (ARSs) are essential enzymes that load amino acids to their cognate tRNA molecules. The expression of certain ARSs and tRNAs has been shown to be deregulated in cancer, presumably to accommodate elevated protein synthesis requirements. In this work, the expression of cytoplasmic ARSs and tRNAs in ten TCGA cancers has been systematically examined. ARSs were found to be mostly upregulated in tumours and their upregulation often correlated with worse patient survival. tRNAs were found to be either upregulated or downregulated in tumours and their expression sometimes correlated to worse survival outcomes. However, although the expression of most ARSs and tRNAs was deregulated in tumours when compared to healthy adjacent tissues, only in a few cases, and independently, did it correlate to patient survival. These data point to the general uncoupling of concomitant ARS and tRNA expression deregulation and patient survival, highlighting the different ARS and tRNA requirements in cancers.

## 1. Introduction

The aminoacyl-tRNA synthetases (ARSs) are essential, ubiquitously expressed enzymes involved in protein translation and conserved from bacteria to humans [1]. They are responsible for the loading of each amino acid to its cognate tRNA molecule, in a two-step aminoacylation reaction process [2]. In the first step, the ARS binds the amino acid and a molecule of ATP to form an aminoacyl adenylate intermediate, while in a second step, the tRNA molecule binds the ARS and the amino acid is transferred to the tRNA [2]. There are 36 ARSs in humans, 17 of which function exclusively in the cytoplasm (AARS, CARS, DARS, EPRS, FARS, HARS, IARS, LARS, MARS, NARS, QARS, RARS, SARS, TARS, VARS, WARS, YARS; Table S1), another 17 in the mitochondria (AARS2, CARS2, DARS2, EARS2, FARS2, HARS2, IARS2, LARS2, MARS2, NARS2, PARS2, RARS2, SARS2, TARS2, VARS2, WARS2, YARS2) and two (GARS and KARS) in both [1,3]. One of the cytoplasmic ARSs, glutamyl-propyl-tRNA synthetase (EPRS), is responsible for charging two cognate tRNAs with either proline or glutamic acid [4]. Eight of the ARSs are found in multiple synthetase complexes together with three scaffolding proteins, the aminoacyl tRNA synthase complex-interacting multifunctional proteins (AIMP1, AIMP2 and AIMP3, the latter also being known as eukaryotic translation elongation factor 1 epsilon 1 or EEF1E1). These are vital for the assembly of the multiple synthetase complexes and their contribution to cellular homeostasis [5].

ARSs have been traditionally viewed as housekeeping genes participating in protein translation, a view that has been changing in the last ten years [6]. More specifically, alter-native functions of ARSs have emerged that highlight their involvement in disease [2,7,8,9,10], including cancer [2,11,12]. Although the mechanistic details are largely unclear, deregulation of ARS expression has been connected to carcinogenesis. For example, methionyl-tRNA synthetase (MARS) and threonyl-tRNA synthetase (TARS) overexpression has been associated with poor clinical outcomes in lung cancer [13] and pancreatic cancer [14], respectively. Tryptophanyl-tRNA synthetase (WARS) is overexpressed in oral squamous cell carcinomas and correlates with tumour stage and invasion [15], whereas several ARSs, including the glycyl(GARS)- and lysyl(KARS)-tRNA synthetases, have been found deregulated in prostate cancer [16].

Similarly to ARSs, the expression of tRNA genes by RNA polymerase III (RNAPIII) has also been considered a housekeeping activity in the past, a view that has gradually changed, as the expression of tRNA genes has been shown to be tightly regulated [17]. tRNA expression has been found overexpressed in cancers in order to sustain increased cell proliferation and growth [18], but specific tRNA gene overexpression has also been reported in skin melanoma [19], multiple myeloma [20] and breast cancer [21]. Moreover, tRNA^iMet^ overexpression has been shown to increase proliferation [22] and tRNA^Glu^ has been shown to directly drive metastasis in breast cancer cells [23].

Although ARSs and tRNAs can be upregulated or downregulated in several different cancers, their relationship has been hypothesised, but not investigated to date. Since the main function of ARSs is the charging of amino acids to their cognate tRNAs, it can be argued that ARS overexpression in cancers would be associated to tRNA overexpression, in order to enhance protein translation. Having said this, it is also possible that individual upregulation or downregulation of ARSs or tRNAs might have no effect on protein translation, but might serve translation-independent functions.

During the last decade, the Cancer Genome Atlas (TCGA) research network, a large collaborative consortium, has accumulated a plethora of cancer data [24]. Thousands of tumour samples from different cancers have been analysed with the same methodologies, both at the DNA and RNA level [24]. It is therefore now possible to question the relationship between ARSs and tRNA overexpression in the same tumour samples in several different cancers. In this work, the open-access TCGA resources were employed to examine the correlation of cytoplasmic ARS and tRNA expression deregulation in patient tumour samples, as well as their link to patient survival.

## 2. Materials and Methods

All the data analysed in this work were generated by the TCGA research network [24]. The data were retrieved from the UCSC Xena hub (https://tcga.xenahubs.net, accessed 1 July 2021) [25] and derived from the TCGA Data Coordinating Center (DCC) in January 2016. Out of all the available TCGA datasets, only 10 contained 30 or more tumour and adjacent normal samples for DNA, mRNA and tRNA analyses, and these were selected for this study (Table S2).

### 2.1. Mutation Number Analysis

The list of cytoplasmic ARS genes was retrieved from the HGNC database [26]. The mutation profiles and DNA alteration frequencies were generated based on TCGA data from the cBioPortal (accessed 26 June 2021) [27,28].

### 2.2. Gene Expression Analysis

The ARS mRNA expression TCGA data were retrieved from the UCSC Xena platform [25]. The tRNA expression TCGA data were originally analysed by Zhang et al. [29] and retrieved from the open-source research platform Synapse (https://www.synapse.org, syn8367012, accessed 15 July 2021). The heatmaps and correlations were computed by Prism v8.4.3 (GraphPad Software, San Diego, CA, USA). The non-parametric Spearman correlation coefficient (r) is provided in each correlation graph. The *p*-values for the tRNA isoacceptor fold change (FC) upregulation were calculated with the unpaired, two-tail, unequal variance *t*-test.

### 2.3. Patient Survival Analysis

The clinical data used in patient survival analyses were retrieved from the UCSC Xena platform [25]. The ARS and tRNA expression and patient survival heatmaps were generated using the UCSC Xena platform [25] and Prism v8.4.3 (GraphPad Software, San Diego, CA, USA). The Cox proportional hazards regression [30] and logrank analyses [31] of the patient survival data were performed by Prism v9.3.1 (GraphPad Software, San Diego, CA, USA).

### 2.4. Statistical Analysis

The statistical analyses were performed by Prism v8.4.3 or v9.3.1 (GraphPad Software, San Diego, CA, USA). The *q*-value (false discovery rate—FDR) was calculated using the Benjamini and Hochberg method [32] in Prism v8.4.3 (GraphPad Software, San Diego, CA, USA). *p* and *q* values < 0.05 were considered significant. The asterisks indicate statistical significance; *p* < 0.05 (*), *p* < 0.01 (**), *p* < 0.001 (***), and *p* < 0.0001 (l). Not significant *p*-values were indicated by “ns”.

## 3. Results

**Figure 1:**
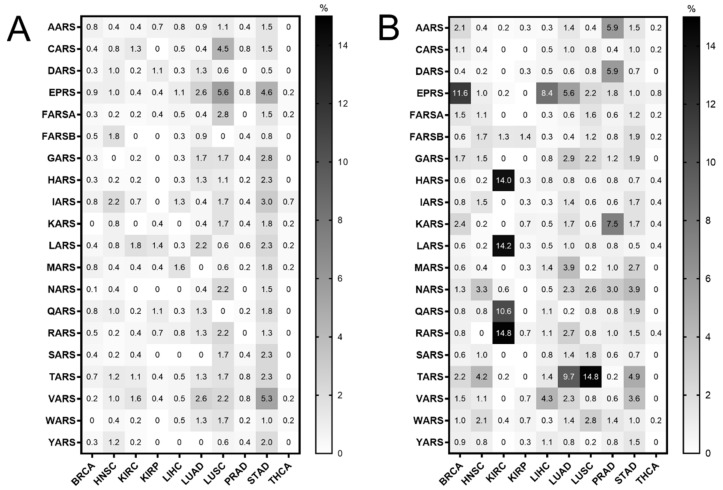
ARS mutational alteration frequencies (%) in TCGA cancers. (**A**) Point mutation alteration frequencies. These include non-silent mutations, such a as nonsense, missense or splice-site introducing mutations. (**B**) Copy number alteration (CNA) frequencies. These include gains/amplifications and losses/deletions.

Mutations in the ARS genes have been associated with human diseases, including cancer [2,11,12]. Therefore, the TCGA cancer data [24] were examined for mutations in ARSs in cancers of different origin. The ARS abbreviations, sample numbers and cancer studies are shown in Tables S1 and S2. The search was narrowed to TCGA studies that contained more than 30 tumour samples and adjacent normal samples for DNA, mRNA and tRNA analyses, to ensure enough samples for valid statistical analyses, and focused on the cytoplasmic ARSs. This led to 10 different cancer datasets that presented ARS mutational alteration frequencies from 0% to 5.6% (Figure 1A). The most mutated ARSs were EPRS in LUSC (5.6%) and STAD (4.6%), VARS in STAD (5.3%) and CARS in LUSC (4.5%). These alterations include non-silent mutations, such a as nonsense, missense or splice-site introducing mutations, as defined in the cBioPortal database [27,28]. As expected, since the ARSs are essential proteins, the overall number of mutations was generally low. Interestingly though, a relatively higher number of structural changes, such as gains/amplifications and losses/deletions, was observed in these genes (Figure 1B). More specifically, the copy number alterations (CNAs) ranged from 0% to 14.8%, with RARS in KIRC (14.8%); TARS in LUSC (14.8%); LARS, HARS and QARS in KIRC (14.2%, 14% and 10.6%, respectively); and EPRS in BRCA (11.6%) most affected. A closer look at the CNAs (Figure S1A,B, respectively) revealed that, overall, there were more amplifications than deletions, resulting in a higher ratio of amplifications versus deletions in most cancers (Figure S1A–C). An examination of the most affected ARSs revealed that apart from QARS in KIRC, CNAs correlated with changes in ARSs gene expression (Figure S2). Increased mutation levels, including CNAs, can be a sign of tumour drivers, genes that are often mutated and contribute to carcinogenesis [33]. It was therefore examined if any of the ARSs had been found to have a potential cancer driver role, using the established cancer driver database Intogen (https://www.intogen.org/, accessed 28 June 2021) [34]. However, according to Intogen [34], none of the ARSs were found to have a cancer driver role in the examined cancers.

**Figure 2:**
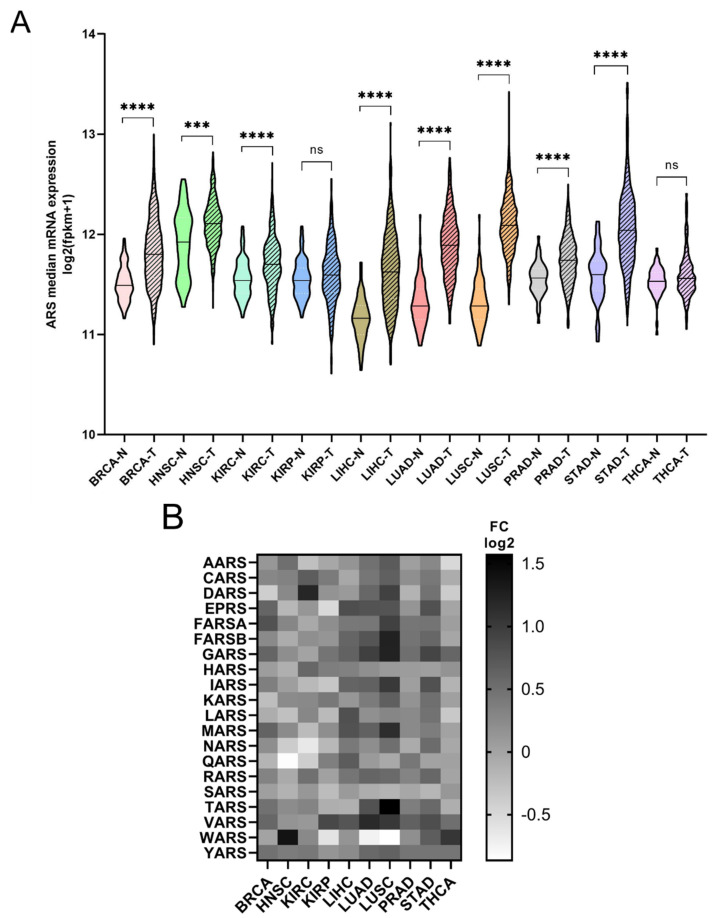
ARS mRNA expression in normal and tumour tissues. (**A**) Median ARS mRNA expression in normal (N) and tumour (T) tissues. The *p*-values were calculated using the non-parametric Mann–Whitney test. **** *p* < 0.0001, *** *p* < 0.001, ns: not significant. (**B**) Fold change (FC) of individual ARS median expression in tumour vs. normal tissues.

The mRNA expression of the ARSs was investigated next. In general, the mRNA expression of ARSs was significantly higher in the tumour samples compared to the adjacent normal tissues, except from KIRP and THCA, which did not achieve statistical significance, despite showing a similar trend to a degree (Figure 2A). The median increase was 19%, with LUSC (75%), LUAD (52%), LIHC (38%) and STAD (36%) showing the highest upregulation. As expected, the ARSs presented differential levels of expression in normal and tumour samples, with VARS increasing its overall median expression among all cancers by 62%, followed by GARS (51%) and YARS (36%) (Figure 2B andFigure S3). Several ARSs were upregulated by more than two-fold, including TARS (3-fold), GARS (2.4-fold), FARSB (2.4-fold each) and MARS (2.2-fold) in LUSC; WARS (2.6-fold) in HNSC; DARS (2.3-fold) in KIRC; MARS (2.3-fold) and VARS (2.2-fold) in LUAD; and YARS (2-fold) in THCA (Figure 2B and Figure S3). Although overall the expression of ARSs increased in tumour samples, the expression of several genes was decreased more than 30%, as compared to the normal samples, with WARS being downregulated in LUSC, LUAD and KIRP (by 45%, 41% and 35%, respectively), as were QARS in HNSC (45%) and NARS in KIRC (36%) (Figure 2B and Figure S3). These data show differential expression regulation of ARSs in tumour samples as compared to those from adjacent healthy tissue.

**Figure 3:**
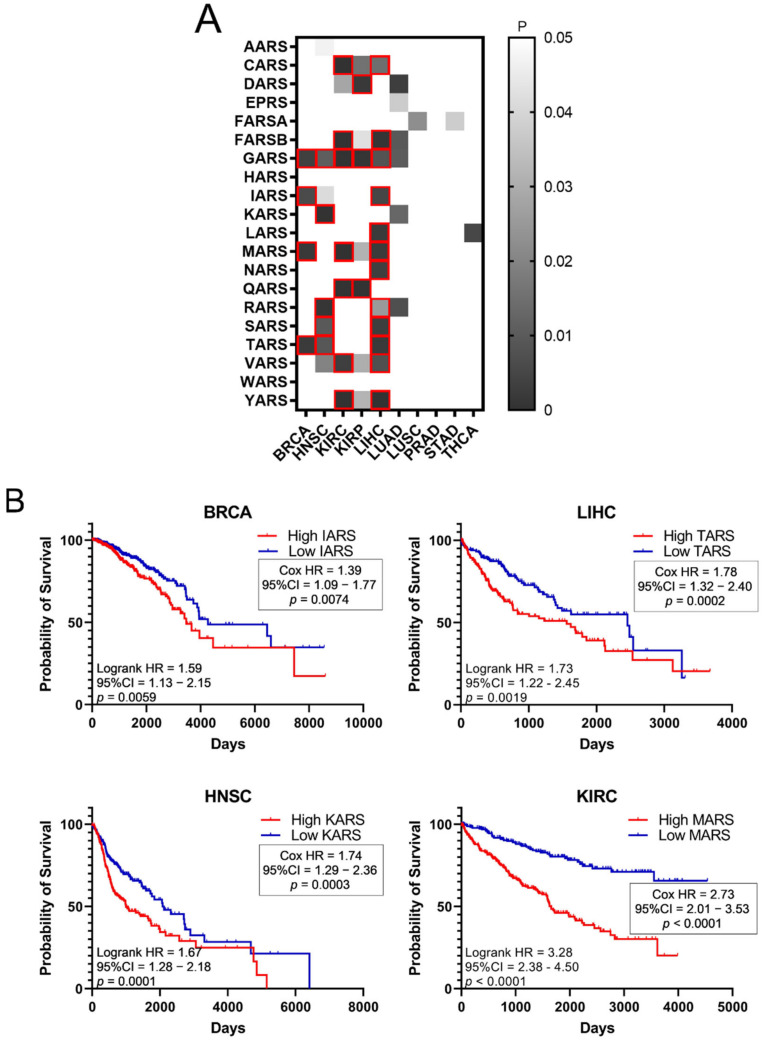
Survival of patients with upregulated or downregulated ARS expression. (**A**) Patient survival *p*-values in tumours with deregulated ARS expression. The grey squares represent *p* < 0.05. The red outlines represent *q* < 0.05, higher ARS expression and worse patient survival. (**B**) Indicative Kaplan–Meier survival plots of patients with tumours presenting upregulated ARS expression.

Higher expression of ARSs in tumours and high fold-change (FC) tumour to normal expression ratios could indicate a potential role of ARSs in cancer. To test the hypothesis that increased ARS expression can affect cancer survival, the survival of patients with elevated ARS expression was tested. To this end, the *p*-values of Kaplan–Meier plots from tumour samples expressing high or low levels of ARSs were plotted as a heatmap (Figure 3A). The grey squares represent *p* < 0.05 (logrank test) and the red outlines represent *p* < 0.05 and *q* < 0.05 for ARSs whose overexpression correlated with worse patient survival. The white squares, seen as white background, represent *p* > 0.05 (not significant). Several ARSs with increased expression correlated with lower patient survival (Figure 3A). As expected, this varied among cancers, with LIHC presenting twelve ARSs correlating with significantly lower survival, followed by KIRC with seven and HNSC with five (Figure 3A). GARS was found to significantly correlate with five different types of cancer tumours (BRCA, HNSC, KIRC, KIRP and LIHC), followed by MARS and TARS that correlated with three different types of tumours (BRCA, KIRC, LIHC and BRCA, HNSC, LIHC, respectively) (Figure 3A). There were no ARSs (*p* < 0.05 and *q* < 0.05) found in which lower expression was associated with worse survival (Figure 3A). Indicative Kaplan–Meier survival plots are shown in Figure 3B, showing that increased expression of certain ARSs correlates with lower patient survival (Figure 3B). These data indicate an important role for ARSs in cancer survival.

**Figure 4:**
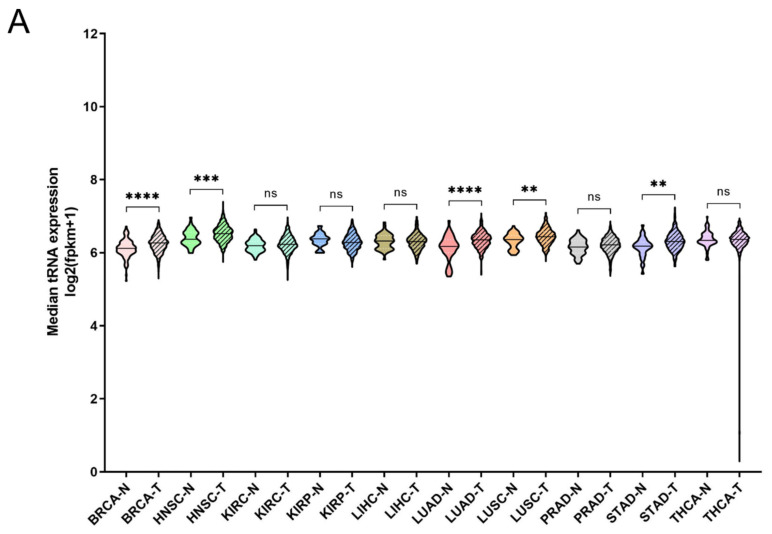

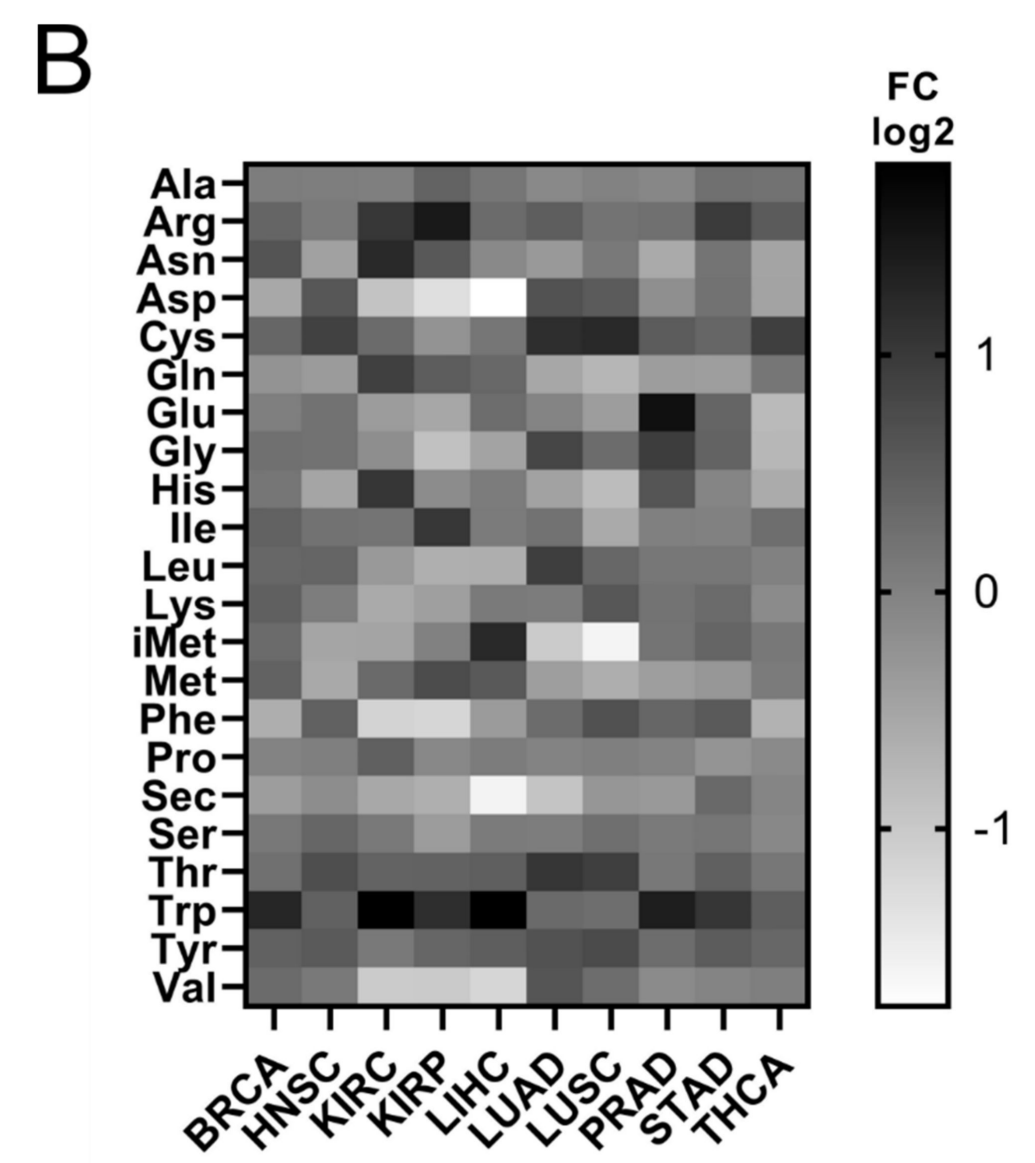
tRNA expression in normal and tumour tissues. (**A**) Median tRNA expression in normal (N) and tumour (T) tissues. The *p*-values were calculated using the Mann–Whitney test. **** *p* < 0.0001, *** *p* < 0.001, ** *p* < 0.01, ns: not significant. (**B**) Fold change (FC) of tRNA median expression in tumour vs. normal tissues.

It has been shown that tRNA expression is altered in certain tumours [21,22,23,35]. It can therefore be argued that increased expression of ARS in tumours might correlate with higher expression levels of the tRNAs to which ARSs bind. To test this hypothesis, the expression of tRNAs in the same 10 cancers and samples was investigated. In only 5 of the 10 cancers the median tRNA expression was significantly upregulated in tumours compared to the adjacent normal tissues (Figure 4A). The overall median increase was 5%, with the most upregulated tRNA expression in LUAD (14%), BRCA (11%) and HNSC (11%). Similarly to ARSs, the tRNAs presented differential levels of expression in normal and tumour samples (Figure 4B and Figure S4). tRNA^Trp^ was the tRNA that was most upregulated in all the examined cancers, with a median of 2.2-fold, followed by tRNA^Cys^ (1.4-fold) and tRNA^Arg^ (1.4-fold) (Figure 4B and Figure S4). tRNA^Trp^ was more than 2-fold upregulated in six cancers (BRCA, KIRC, KIRP, LIHC, PRAD and STAD), tRNA^Cys^ in two (LUAD and LUSC) and tRNA^Arg^ also in two (KIRC and KIRP) (Figure 4B and Figure S4). In contrast, several tRNAs were downregulated in the tumour samples. tRNA^Sec^, tRNA^Asp^ and tRNA^Gln^ showed a median decrease of 22%, 20%, and 19%, respectively, being downregulated in several cancers (Figure 4B and Figure S4). It is important to note that several tRNAs presented a more than 50% decrease in their expression in tumours, with tRNA^Asp^ downregulated in LIHC and KIRC by 70% and 60%, respectively; tRNA^Val^ in LIHC (56%), KIRC (51%) and KIRP (51%); tRNA^Phe^ in KIRP (56%) and KIRC (55%); tRNA^iMet^ in LUSC (67%) and LUAD (51%); and tRNA^Sec^ in LIHC (67%) (Figure 4B and Figure S4). These data indicate that the tRNA isotype expression differs in different cancers and can be upregulated or downregulated.

**Figure 5:**
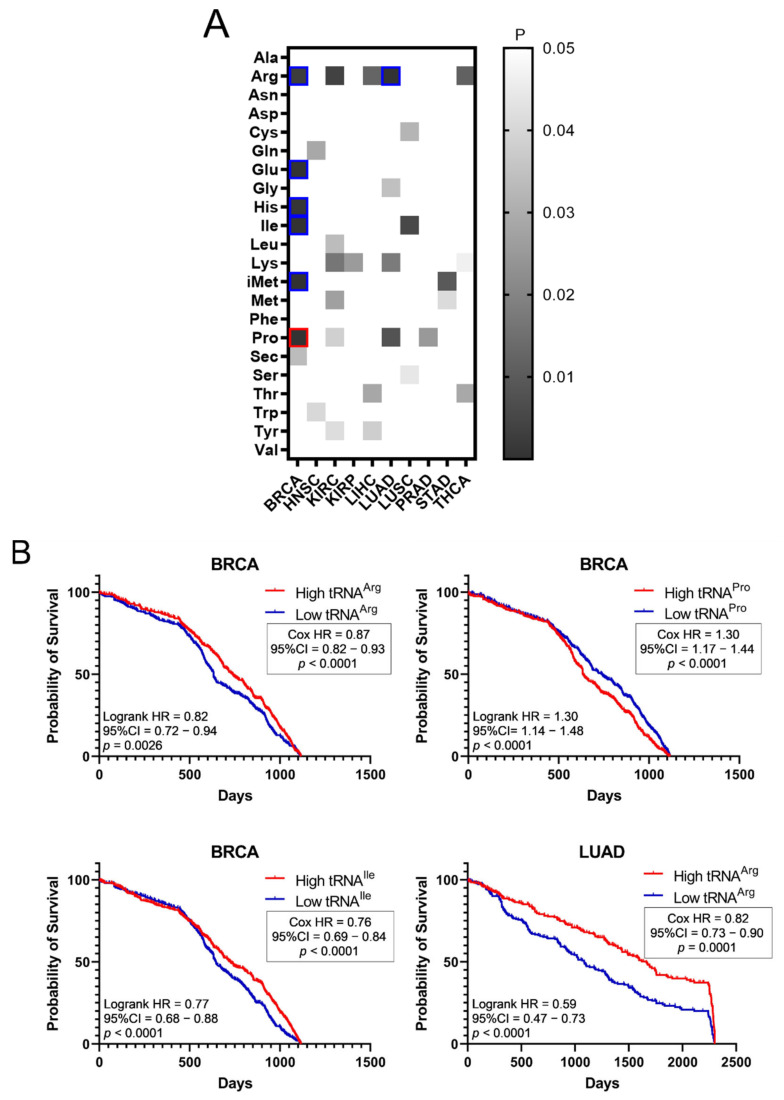
Survival of patients with upregulated or downregulated tRNA expression. (**A**) Patient survival *p*-values (logrank test) in tumours with upregulated or downregulated tRNA expression. The grey squares represent *p* < 0.05. The red outlines represent *q* < 0.05 for tRNAs whose overexpression correlated with worse patient survival, while the blue outlines represent *q* < 0.05 for tRNAs whose downregulation correlated with worse patient survival. (**B**) Indicative Kaplan–Meier survival plots of patients with tumours presenting upregulated or downregulated tRNA expression.

The effect of tRNA expression on patient survival was investigated next (Figure 5). The aim was to examine if higher or lower tRNA expression in tumours is correlated with patient survival. The *p*-values of Kaplan–Meier plots from tumour samples expressing high or low levels of tRNAs were plotted as a heatmap (Figure 5A). The grey squares represent *p* < 0.05, the red outlines represent *p* < 0.05 and *q* < 0.05 for tRNAs whose overexpression correlated with worse patient survival, while the blue outlines represent *p* < 0.05 and *q* < 0.05 for tRNAs whose downregulation correlated with worse patient survival (Figure 5A). It was found that higher expression of tRNA^Pro^ in BRCA correlated with worse patient outcomes, whereas decreased expression of tRNA^Arg^, tRNA^Glu^, tRNA^His^, tRNA^Ile^ and tRNA^iMet^ in BRCA, as well as tRNA^Arg^ in LUAD, correlated with worse patient outcomes (Figure 5A). Indicative patient survival plots for the above are shown in Figure 5B. These data collectively reveal that either upregulation or downregulation of tRNA expression in tumours can correlate with worse patient survival outcomes.

**Figure 6:**
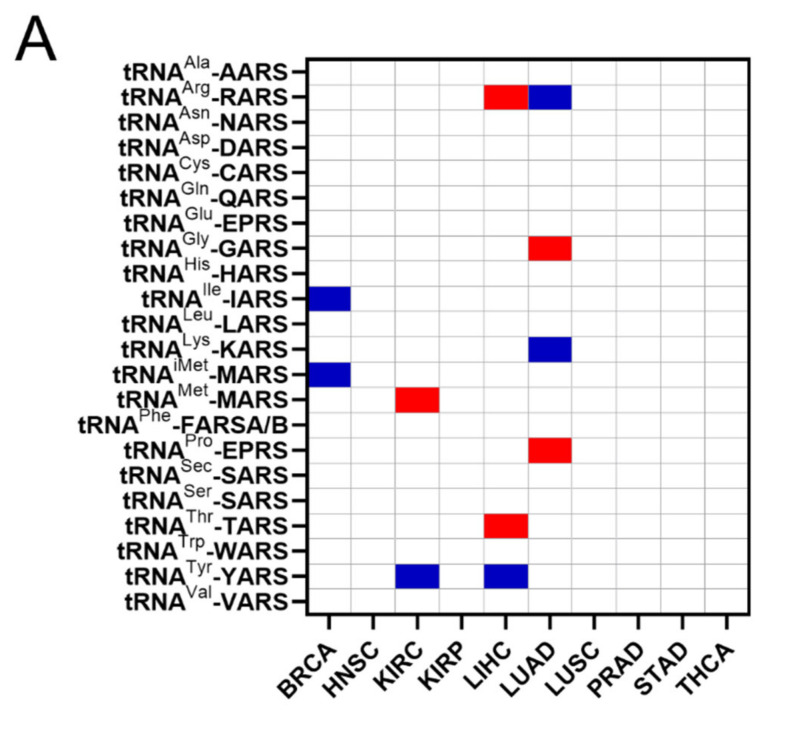

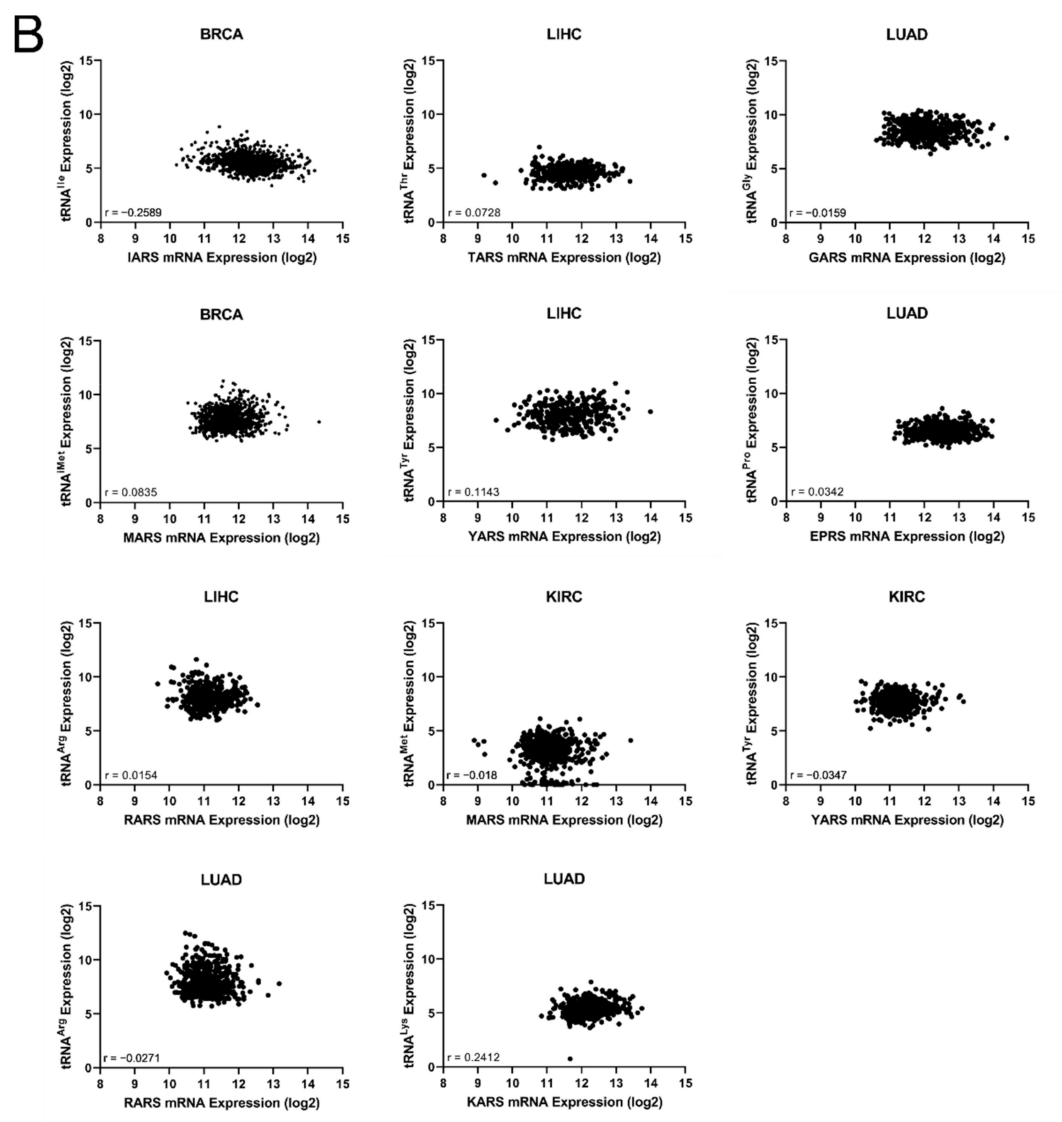
Concomitant ARS and tRNA expression deregulation in tumours. (**A**) Concomitant deregulation of ARS and tRNA expression in tumours affecting patient survival. Red squares denote ARS and tRNA overexpression associated with worse patient survival, while blue squares denote upregulation of ARS, but downregulation of their cognate tRNA expression and association with worse patient survival. This panel was generated based on the *p*-values of the data shown in Figure 3A and Figure 5A. (**B**) Correlation of ARSs mRNA and their cognate tRNA expression. The Spearman correlation coefficient (r) is shown.

The common occurrences where both ARSs and tRNA expression deregulation correlated with patient survival were then determined (Figure 6A). More specifically, there were eleven cases found in total, with a *p*-value < 0.05 for both ARSs and tRNAs. In five of those, overexpression of ARSs and their cognate tRNAs was related with worse patient survival (shown in red, Figure 6A), and in six cases overexpression of ARSs but downregulation of their cognate tRNAs was related with worse patient survival (shown in blue, Figure 6A). It is important to note that for some of these cases, although the *p*-values were <0.05, this was not the case for the *q*-values (Figure 3A and Figure 5A), suggesting a higher probability of being false positives. If the observed concomitant expression deregulation of ARSs and tRNAs was associated with patient survival, a correlation between them might have been expected within each tumour. However, there was no correlation observed between ARS mRNA and tRNA expression (Figure 6B). These data suggest that although in some cases both the ARS and its cognate tRNA deregulation were associated with patient survival, there was no correlation, positive or negative, found between their RNA expression.

**Figure 7:**
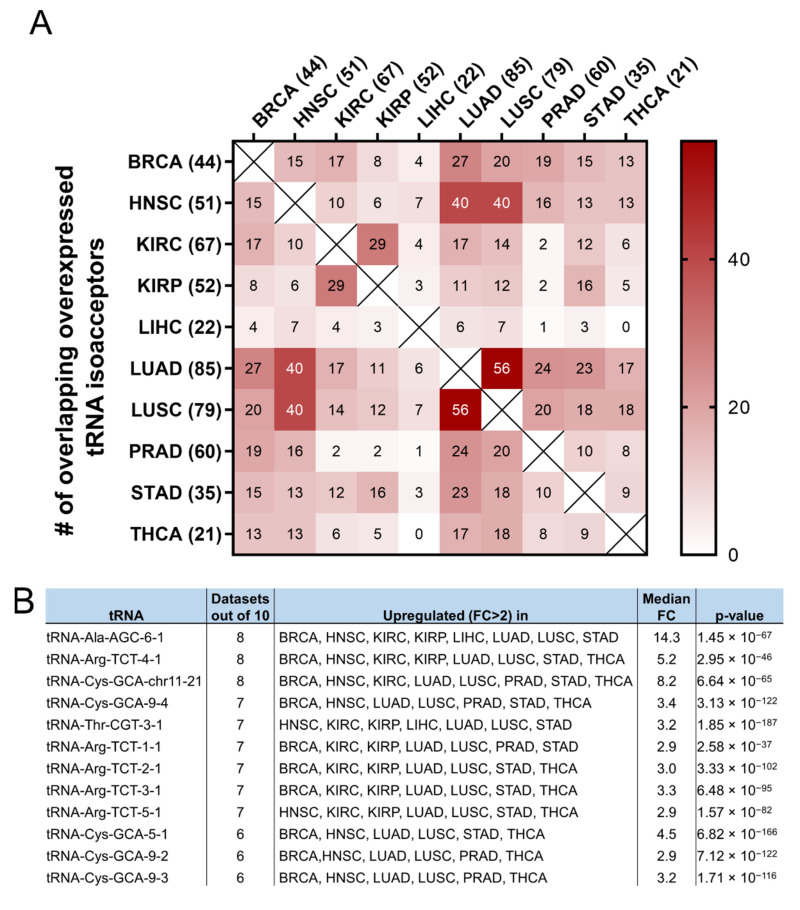

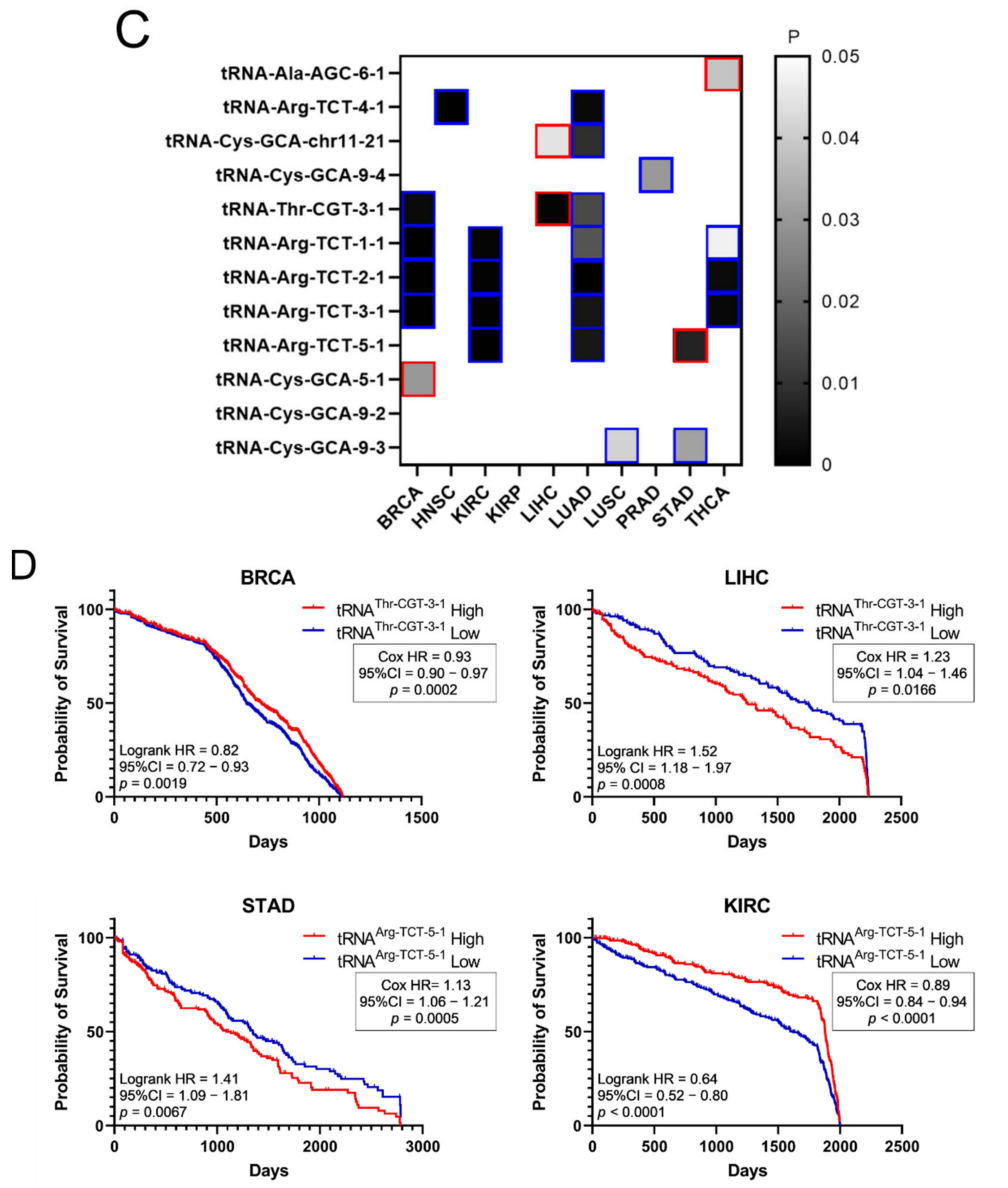
tRNA isoacceptor expression. (**A**) common tRNA isoacceptor upregulation (FC > 2, *p* < 0.05 and *q* < 0.05) in different TCGA cancers. The *p*-values were calculated with the two-tail, unequal variance *t*-test. (**B**) Most common upregulated tRNA isoacceptors. The fold change (FC) shows upregulation in the tumour (5170) vs. the normal (553) samples in all datasets. The *p*-values were calculated with the unpaired, two-tail, unequal variance *t*-test. (**C**) Patient survival *p*-values in tumours with upregulated (red outlines) or downregulated (blue outlines) tRNA isoacceptor expression that correlated with worse patient survival. The grey squares represent *p* < 0.05 and *q* < 0.05. (**D**) Indicative Kaplan–Meier survival plots of patients with tumours presenting upregulated or downregulated tRNA isoacceptor expression.

It was then investigated if the tRNA specific isoacceptor expression was better associated with patient survival than the tRNA isotype expression. To this end, the expression of individual tRNA isoacceptors that were deregulated by more than two-fold (FC > 2, *p* < 0.05, *q* < 0.05) in cancer, compared to the adjacent healthy tissue, was investigated. It was found that many tRNA isoacceptors are consistently upregulated in different cancers (Figure 7A). For example, 56 out of the 85 tRNA isoacceptors overexpressed in LUAD were also overexpressed in LUSC (66%) and 40 in HNSC (47%) (Figure 7A). Intriguingly, several tRNA isoacceptors were found overexpressed in most of the examined datasets (Figure 7B). These include tRNA^Ala-AGC−6−1^, tRNA^Arg-TCT−4−1^ and tRNA^Cys-GCA-chr11−21^, which were significantly overexpressed more than 5-fold in 8 out of the 10 examined cancers (Figure 7B). However, similar to the tRNA isotype expression, the upregulation of these tRNA isoacceptors in the tumours as compared to normal tissue did not necessarily correlate with worse patient survival. Indeed, upregulation (red) or downregulation (blue) of these tRNA isoacceptors’ expression correlated with worse patient survival (Figure 7C,D), revealing no clear connection between tRNA isoacceptor expression deregulation in the tumours and patient survival.

## 4. Discussion

Deregulation of ARS expression has previously been implicated in disease and especially in cancer [2,11,12]. Similarly, tRNA overexpression has been previously associated with cancer progression [18,21,23]. In this work, unbiased genomic approaches were employed to investigate the relationships between ARS mRNA and tRNA expression and patient survival in cancers of different origin. No general patterns were revealed for either the ARSs or the tRNAs. The ARS mutation frequencies were relatively low and inconsistent, while their expression varied among cancers, as did the expression of tRNAs. Overexpression of certain ARSs strongly correlated with decreased patient survival in some cancers, but not with concomitant overexpression of their cognate tRNAs. tRNAs were found to be upregulated or downregulated in tumours and, in contrast to ARSs, both their upregulation and downregulation correlated with decreased patient survival.

The ARSs were mutated in several cancers (Figure 1A,B). However, they are essential enzymes, and as such it is not a surprise that their alteration frequencies overall are low (Figure 1A,B), with most of the selected mutations being copy number gains (amplifications) (Figure 1B and Figure S1). Chromosomal gains/amplifications of certain ARSs were found in several cancers, with HARS, LARS and RARS in KIRC, EPRS in BRCA and TARS in LUSC at relatively high frequencies B and Figure S1). Interestingly, the mRNA expression of ARSs affected by amplifications in the tumours examined was increased (Figure S2), suggesting that many of these gains/amplifications at the DNA level were actively transcribed and contributed to the elevated mRNA expression. There were fewer losses/deletions of ARSs observed (Figure S1B,C), with the most pronounced one being QARS in KIRC. Interestingly though, in that case the median QARS mRNA expression did not seem to significantly change in the tumours with QARS losses as compared to the ones without (Figure S2A). It is not clear why this is the case, but it might be attributed to partial DNA loss or decreased mRNA degradation. Although significant overexpression of certain genes, namely cancer drivers, can drive carcinogenesis, none of the ARSs were found to be a potential cancer driver gene [34], in accordance with a previously published report on amplification-dependent cancer driver genes in TCGA cancers [36].

Regardless of the alteration frequencies at the DNA level, the mRNA expression of most ARSs was upregulated in the examined cancers (Figure 2A). This is in general agreement with a recent study that also found that most ARSs were upregulated independently of the DNA alteration frequencies [37]. More specifically, in eight of the ten cancers, the median ARS mRNA expression was significantly upregulated (Figure 2A). It is important to note that in most cases the mRNA expression of ARSs varied significantly among them and between normal and tumour samples (Figure 2B, Figures S3 and S5A), with VARS being the most upregulated ARS mRNA among all tumours, as compared to normal adjacent tissues, followed by GARS and TARS (Figure 2B).

Importantly, upregulation of ARSs broadly correlated with worse outcomes of patient survival (Figure 3A). For example, upregulation of GARS correlated with worse patient survival in BRCA, HNSC, KIRC, KIRP and LIHC, as did upregulation of MARS in BRCA, KIRC and LIHC, and TARS in BRCA, HNSC and LIHC (Figure 3A,B). Upregulation of CARS, FARSB, IARS, QARS, RARS, SARS, VARS and YARS mRNA also correlated with lower patient survival in more than one cancer (Figure 3A). However, not all ARS overexpression correlated with worse patient survival outcomes. Statistical significance (*p* < 0.05, *q* < 0.05) was not reached for some cancers, such as LUAD, LUSC, PRAD, STAD and THCA, although, apart from PRAD, they presented at least one ARS with which its overexpression correlated with decreased patient survival (*p* < 0.05, but *q* > 0.05) (Figure 3A). Similar conclusions were reached by a recent study that followed different research methodologies and included the mitochondrial ARSs as well as the AIMPs [37]. Taken together, the above data indicate that it is not the overall overexpression of ARSs that is related to patients’ survival, but rather the overexpression of specific ARSs in specific tumours. It is of interest to note that based on these observations, the expression of certain ARSs could be of future prognostic value for patient survival, after they become validated by experiments designed to confirm the potential biomarker value of these ARSs.

The deregulation of tRNAs in individual tumours and cancer cell lines has already been established [18,38]. The expression of tRNAs in 10 TCGA cancers was systematically evaluated in this work. The median tRNA expression was significantly altered in five out of the ten cancers (Figure 4A). Among them, the median tRNA expression was upregulated in BRCA, an observation in accordance with previous studies reporting overexpression of tRNAs in breast cancer cell lines and tumours [21,29,39]. Not much is known about the contribution of tRNA deregulation in cancers other than of the breast, but the data in this work suggest that it might play a significant role in HNSC, LUAD, LUSC and STAD (Figure 4A). It is important to note that the tRNA isolated and sequenced by the TCGA consortium does not necessarily accurately represent the full repertoire of tRNAs at a given time. Due to their structure and post-transcriptional modifications, tRNAs can present significant difficulties during their processing for small RNA sequencing. Several novel methods for tRNA isolation and sequencing have been presented recently [40,41,42,43,44,45]. Although these methods are more accurate at representing tRNA expression, they have not to our knowledge been used to isolate tRNAs from tumours and adjacent normal tissues. Therefore, in our view, the current data from the TCGA project, with their inherent vulnerabilities, are the best currently existing that allow multi-omic analyses to be performed, enabling the analysis of ARS/tRNA expression in different cancers and the correlation of gene expression with patient survival.

Interestingly, individual tRNA isotype expression in most cases could significantly be either upregulated or downregulated in tumours when compared to the normal control tissue (Figure 4B, Figures S4 and S5B). tRNA^Trp^, tRNA^Cys^ and tRNA^Arg^ were the most upregulated, while tRNA^Gln^, tRNA^Met^ and tRNA^Sec^ were often, but not always, downregulated (Figure 4B). Importantly it was found that the expression of tRNA^iMet^, the tRNA responsible for transferring the initiator methionine (iMet), was upregulated in breast cancer (Figure 4B). Upregulation of tRNA^iMet^ in breast cells has been reported before and linked to tumour initiation [21], while tRNA^iMet^ has also been shown to promote cancer cell migration and invasion [18], as well as tumour growth and angiogenesis [46]. Based on the data presented in this work (Figure 4B), tRNA^iMet^ was also upregulated in LIHC and, to a lesser degree, STAD, and therefore it would be interesting to investigate the role of tRNA^iMet^ in these cancers.

The upregulation or downregulation of individual tRNA expression correlated with worse patient survival (Figure 5A,B). For example, upregulation of tRNA^Pro^ expression in BRCA correlated with worse patient survival, while the downregulation of five other tRNAs correlated with worse patient survival (Figure 5A,B). This is in contrast with ARS overexpression, which was found to correlate exclusively with worse patient survival (Figure 3). Importantly, concomitant deregulation of RNA expression of both the ARS and its cognate tRNA was observed only in 11 cases, in 5 of which the upregulation of tRNA expression correlated with worse patient survival, while in the other 6 the tRNA downregulation correlated with worse patient survival (Figure 6A). Moreover, there was no significant correlation found between the ARS mRNA expression and the tRNA expression (Figure 6B), as could be expected if their co-expression was of functional significance. The above data suggest that the concomitant deregulation of ARS mRNA and tRNA expression is not essential for their functional contribution to patient survival.

Following from the above, the possibility that specific tRNA isoacceptors, rather than isotypes, associate better with patient survival was investigated. Many of the same isoacceptors were found to be consistently upregulated in several of the examined cancers (Figure 7A) and it is envisaged that some of them could be used as potential cancer biomarkers (Figure 7B), something that could be experimentally validated in future studies. The expression of specific isoacceptors has been shown to have a role in breast cancer metastasis [23], translation regulation [35] and more recently in stress-induced tRNA fragmentation [47], which in turn can repress protein translation and cell growth [48]. Therefore, the finding that many isoacceptors (Figure 7A,B) are commonly overexpressed in different cancers might indicate a role in specific stages of carcinogenesis and/or stress-induced responses, affecting protein translation and cell growth.

As there was no significant correlation found between the tRNA isotypes and their ARSs (Figure 6B), it was investigated if the expression of individual isoacceptors and the expression of their relevant ARSs were positively or negatively correlated. However, similarly to the findings in Figure 6B, no strong or moderate correlations were detected, at least for the isoacceptors shown in Figure 7B, in BRCA and LUAD (Figures S6 and S7). Among the twelve tRNA isoacceptors examined, tRNA^Thr-CGT−3−1^ and perhaps tRNA^Ala-AGC−6−1^ were marginally better correlated with their respective ARSs (Figures S6 and S7). It is probable that the expression of certain tRNA isoacceptors is better correlated with their relevant ARSs, rather than the isotypes, but these isoacceptors might not necessarily be as highly overexpressed as the ones examined in this work (Figure 7B).

Similarly to the tRNA isotype expression (Figure 5A), both the upregulation and downregulation of the tRNA isoacceptors examined (Figure 7B) were found to correlate with decreased patient survival (*p* < 0.05, *q* < 0.05, Figure 7C,D). In contrast, only ARS overexpression was associated with lower patient survival (Figure 3A). Consequently, there seems to be an uncoupling between ARS/tRNA expression and patient survival in cancer, which was underlined by the finding that the tRNA (isotype or isoacceptor) and ARS mRNA expression were mostly uncoupled (Figure 6B, Figures S6 and S7). 

The data on tRNA isotypes and isoacceptors presented in this work indicate that the individual deregulation of ARS mRNA and tRNA expression might affect carcinogenesis and patient survival in different ways. The overall upregulation on ARS expression in cancers is likely a functional adaptation to the increased need of protein translation in cancer cells. However, the specific high overexpression of individual ARSs in certain cancers implies that these ARSs could have roles in cancer, independently of aminoacylation [2,8]. Regarding the tRNA expression deregulation, it has been previously shown that different subsets of tRNAs are favoured in cell proliferation versus cell differentiation [35]. For example, in the case of BRCA, while tRNA^iMet^ overexpression might significantly contribute to tumour initiation, at the later stages of tumourigenesis it is the upregulation of isoacceptors tRNA^Arg-CCG^ and tRNA^Glu-UUC^ that will promote metastasis [23]. It is therefore probable that cancers benefit not only from the overall overexpression of ARSs and tRNAs needed for increased protein synthesis, but also from the specific imbalance in the ratios of ARS and cognate tRNAs, resulting either in the mischarging of tRNAs, leading to translational errors [49,50], or the preferential recruitment of specific tRNA pools that could affect translational speed and efficiency [23,51].

To summarise, this work investigated the relationships between ARS mRNA and tRNA expression and patient survival in ten cancers. Overexpression of specific ARSs strongly correlated with decreased patient survival in some cancers, but not with concomitant overexpression of their cognate tRNAs. tRNAs were found to be upregulated or downregulated in tumours and, contrary to ARSs, both their upregulation and downregulation correlated with decreased patient survival. Although the expression of specific tRNA isoacceptors varies in different cancers, certain isoacceptors were upregulated in most cancers examined, presumably to ensure high levels of protein translation, cell growth and cancer progression.

## Data Availability

The data reported in this work are available from the author upon request. The original data examined in the study can be retrieved from the following sources: UCSC Xena (https://tcga.xenahubs.net, accessed 1 July 2021), cBioPortal (http://www.cbioportal.org/, accessed 26 June 2021) and Synapse (Synapse (https://www.synapse.org, syn8367012, accessed 15 July 2021).

## Supplementary Materials

The following are available online at https://www.mdpi.com/article/10.3390/cimb44070207/s1: Figure S1: Copy number alterations (CNAs) in ARSs; Figure S2: mRNA expression of samples with or without CNAs; Figure S3: mRNA expression of ARS genes in normal tissues (N) or tumours (T) in 10 TCGA cancers; Figure S4: tRNA expression in normal tissues (N) or tumours (T) in 10 TCGA cancers; Figure S5: ARS and tRNA Fold-Change (FC) q-values in cancers; Figure S6: ARS and tRNA isoacceptor expression correlations in BRCA; Figure S7: ARS and tRNA isoacceptor expression correlations in LUAD; Table S1: Aminoacyl-tRNA synthetases; Table S2: TCGA cancer types and sample numbers per TCGA dataset.
