# Supplementary material for: The Aminoacyl-tRNA Synthetase and tRNA Expression Levels Are Deregulated in Cancer and Correlate Independently with Patient Survival
Source: Curr Issues Mol Biol. 2022 Jul 2;44(7):3001–17. doi: 10.3390/cimb44070207 (PMC9324904; doi:10.3390/cimb44070207)
Supplement: Supplementary file 1 [file cimb-44-00207-s001.zip › Figures S1-S7_Revised.pdf]

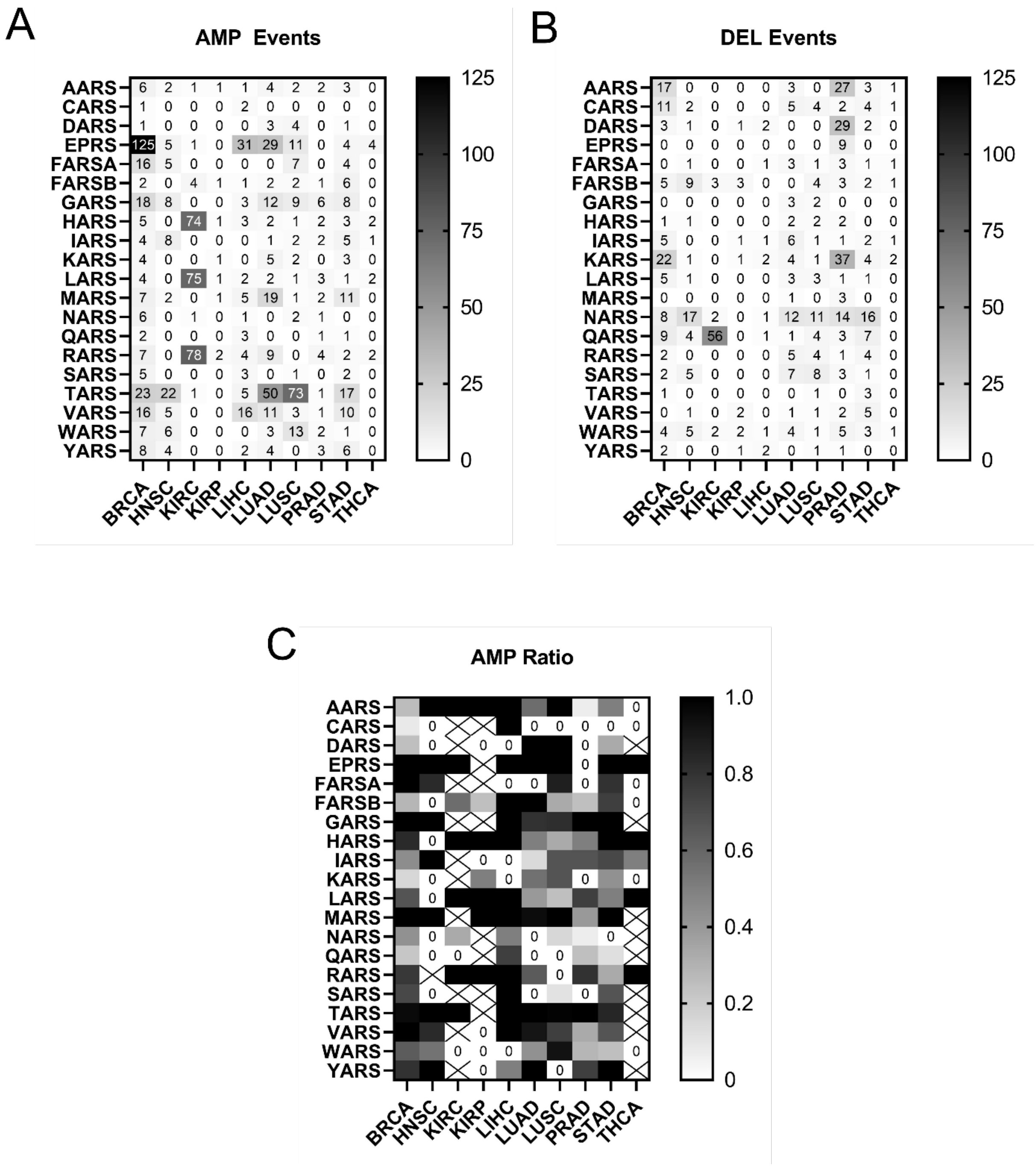

Figure S1. Copy number alterations (CNAs) in ARSs. (**A**) Amplification (AMP) events in ARSs. The numbers represent the events. (**B**) Deletion (DEL) events in ARSs. The numbers represent the events. (**C**) Amplification ratio. The ratio represents the number of amplification events divided by the total number of alterations (amplifications and deletions). For example, an AMP ratio of 0.8 denotes that 80% of the CNAs were amplifications and 20% deletions. 0 denotes no amplifications events, X denotes no amplification or deletion events.

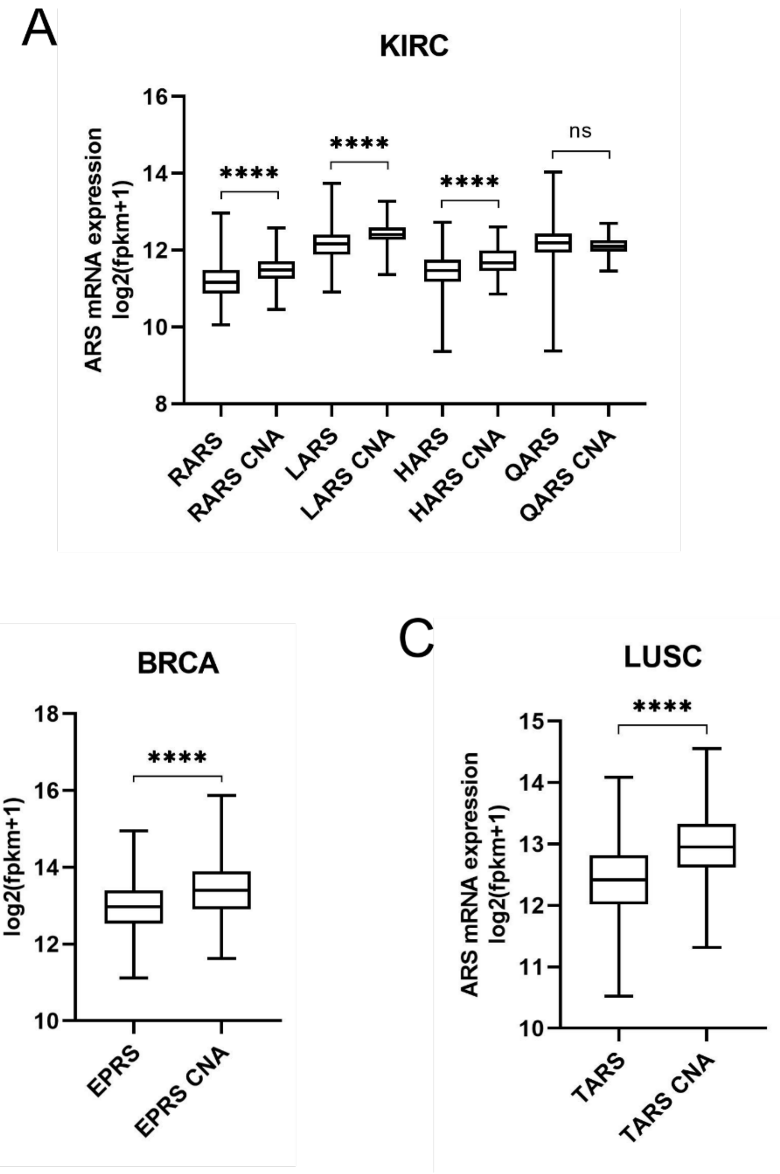

Figure S2. mRNA expression of samples with or without CNAs. The mRNA expression of ARSs with CNAs was compared to those without. (**A**) ARS CNAs and mRNA expression in KIRC. The p-values were calculated by the Mann-Whitney test. (**B**) EPRS CNAs and mRNA expression in BRCA. The p-value was calculated by the Mann-Whitney test. (**C**) TARS CNAs and mRNA expression in LUSC. The p-value was calculated by the Mann-Whitney test. $p<0.0001$ (****), ns: not significant.

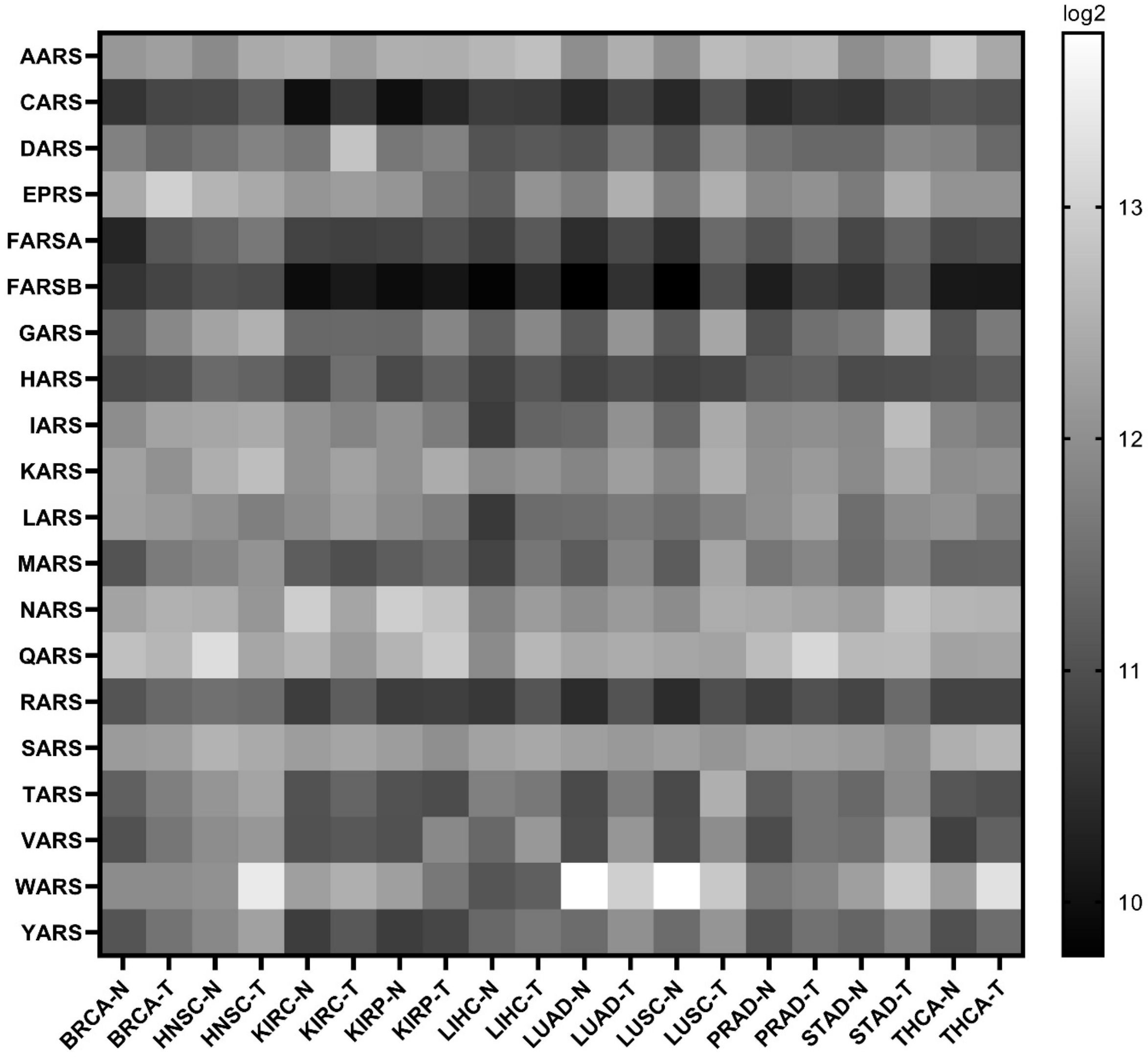

Figure S3. mRNA expression of ARS genes in normal tissues (N) or tumours (T) in 10 TCGA cancers.

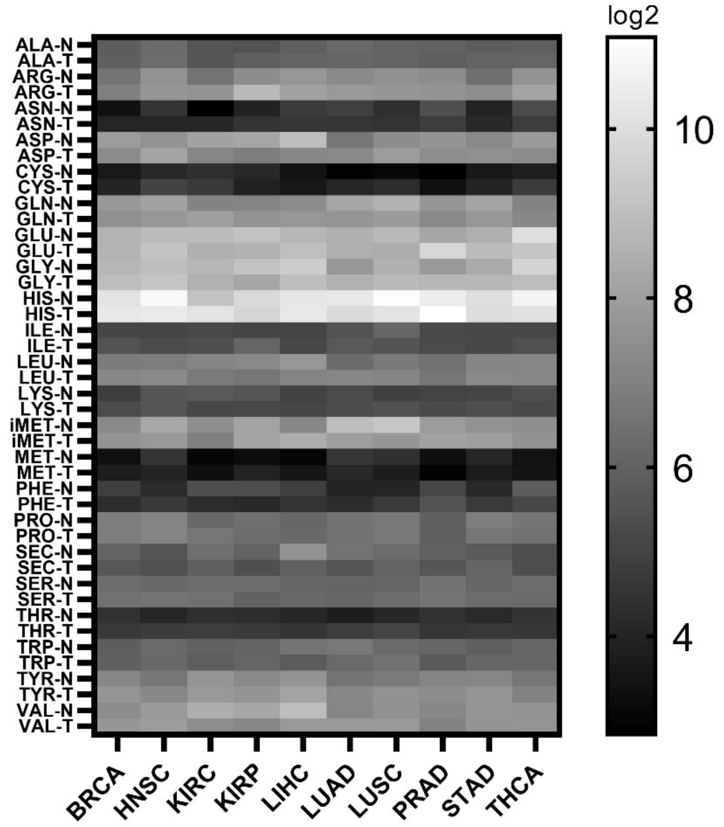

Figure S4. tRNA expression in normal tissues (N) or tumours (T) in 10 TCGA cancers.

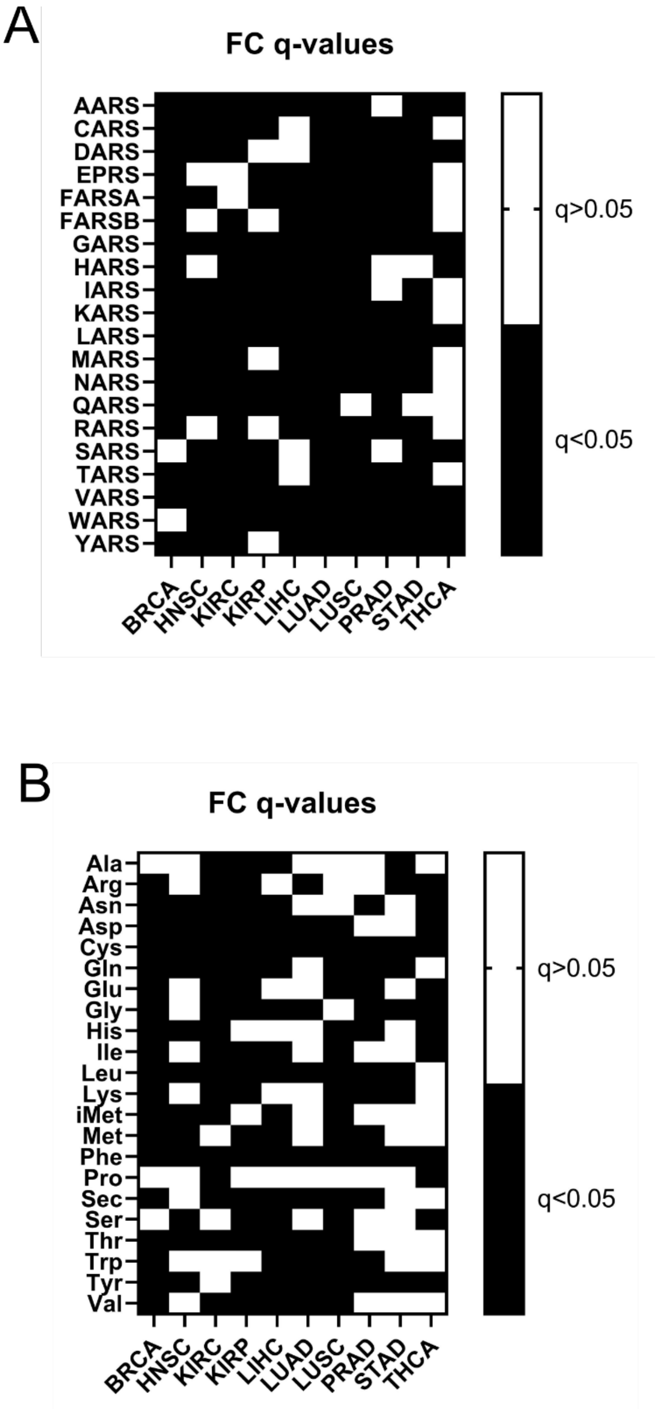

Figure S5. ARS and tRNA Fold-Change (FC) q-values in cancers. (**A**) Significant (black) or not (white) q-values for the ARS gene expression FCs in tumour versus normal tissues. This figure is related to figures 2B and S3. (**B**) Significant (black) or not (white) q-values for the tRNA gene expression FCs in tumour versus normal tissues. This figure is related to figures 4B and S4. The q-values were calculated using the Benjamini and Hochberg method. $q<0.05$ was considered significant.

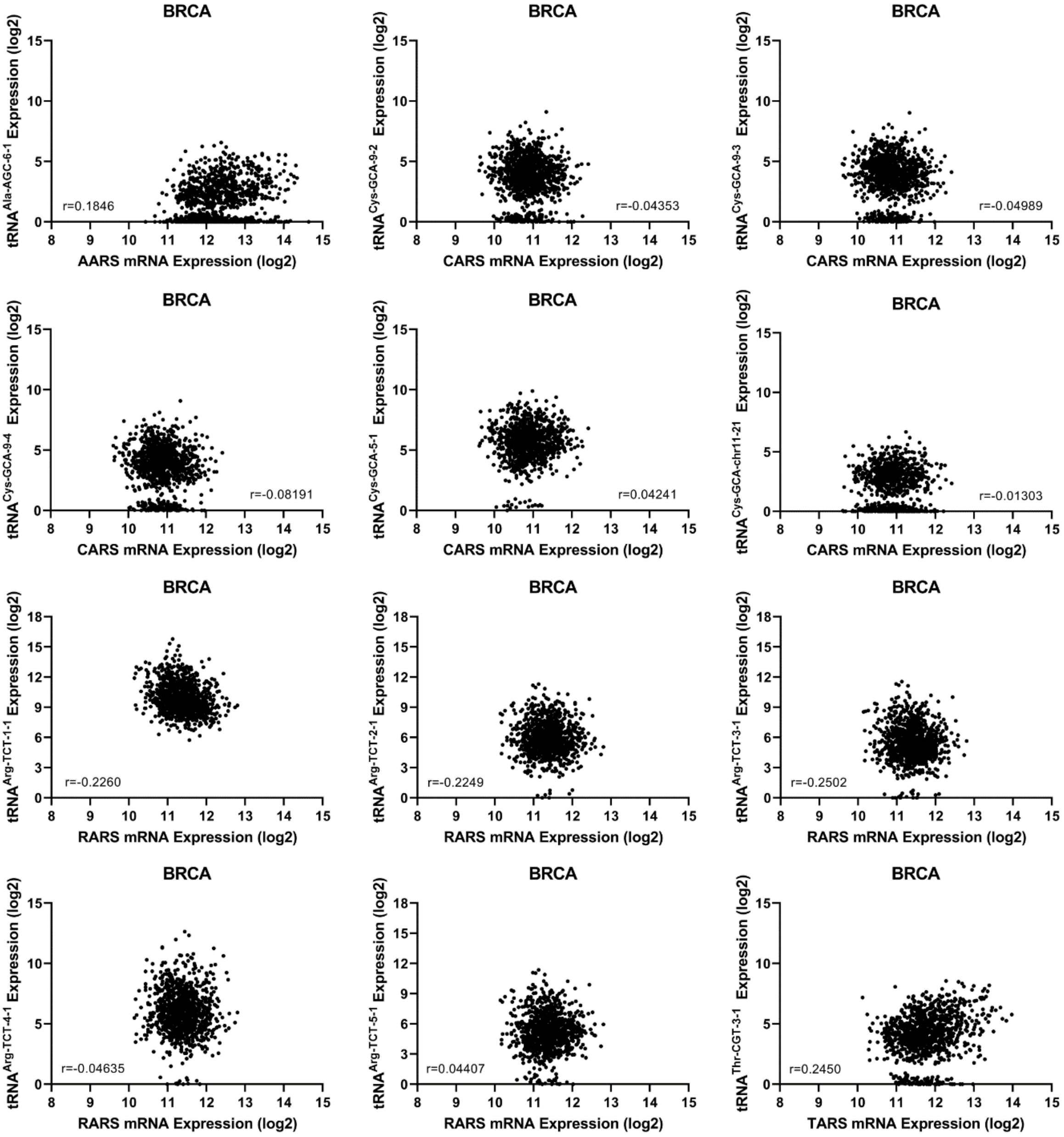

Figure S6. ARS and tRNA isoacceptor expression correlations in BRCA. This figure relates to figure 7B.

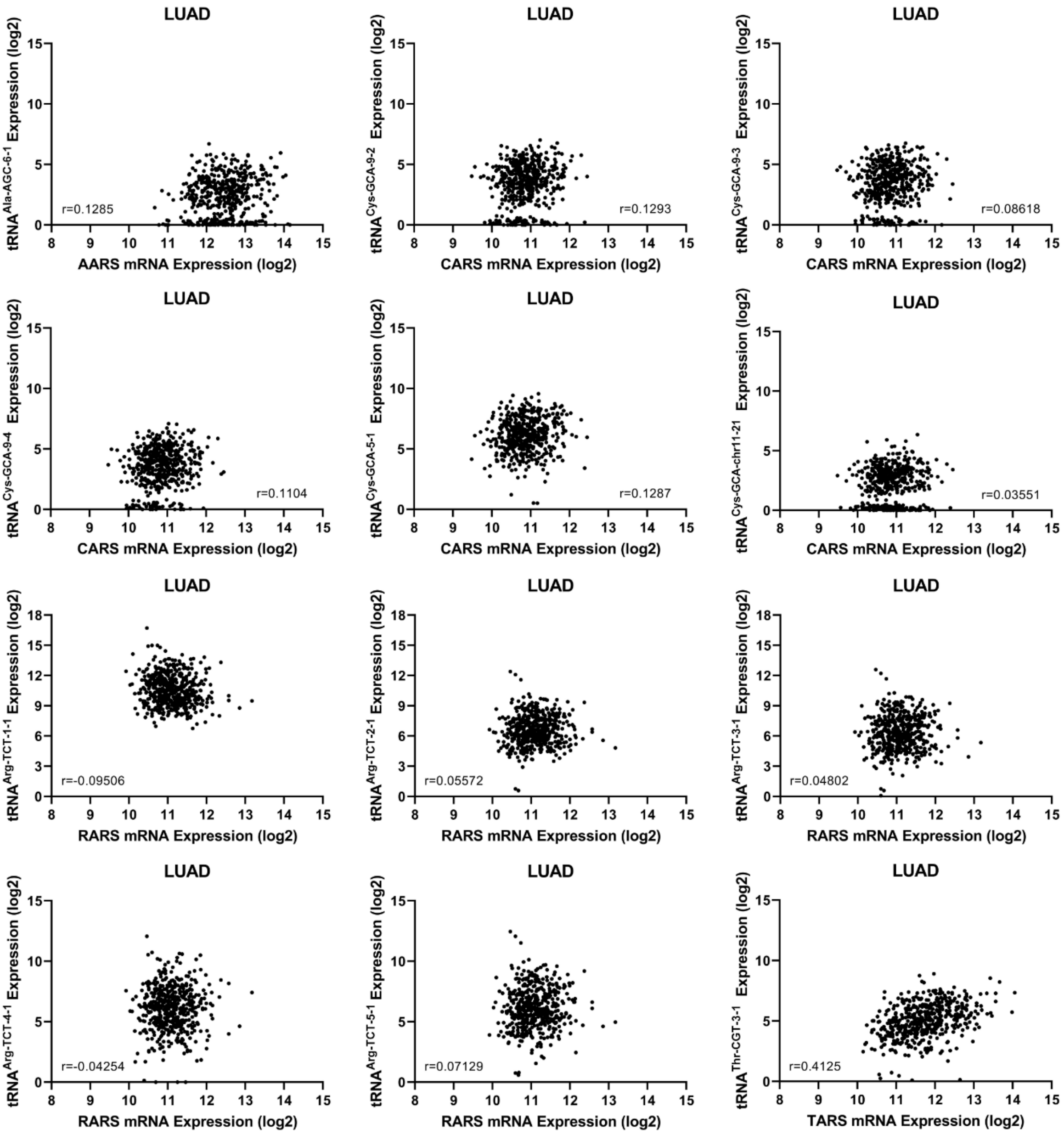

Figure S7. ARS and tRNA isoacceptor expression correlations in LUAD. This figure relates to figure 7B.
