# Supplementary material for: The Aminoacyl-tRNA Synthetase and tRNA Expression Levels Are Deregulated in Cancer and Correlate Independently with Patient Survival
Source: Curr Issues Mol Biol. 2022 Jul 2;44(7):3001–17. doi: 10.3390/cimb44070207 (PMC9324904; doi:10.3390/cimb44070207)
Supplement: Supplementary file 1 [file cimb-44-00207-s001.zip › Tables S1-S2-Revised.pdf]

**Table S1.** Aminoacyl-tRNA synthetases

| Symbol | Name |
|---|---|
| **AARS** | Alanyl-tRNA synthetase |
| **CARS** | Cysteinyl-tRNA synthetase |
| **DARS** | Aspartyl-tRNA synthetase |
| **EPRS** | Glutamyl-prolyl-tRNA synthetase |
| **FARSA** | Phenylalanyl-tRNA synthetase subunit alpha |
| **FARSB** | Phenylalanyl-tRNA synthetase subunit beta |
| **GARS** | Glycyl-tRNA synthetase |
| **HARS** | Histidyl-tRNA synthetase |
| **IARS** | Isoleucyl-tRNA synthetase |
| **KARS** | Lysyl-tRNA synthetase |
| **LARS** | Leucyl-tRNA synthetase |
| **MARS** | Methionyl-tRNA synthetase |
| **NARS** | Asparaginyl-tRNA synthetase |
| **QARS** | Glutaminyl-tRNA synthetase |
| **RARS** | Arginyl-tRNA synthetase |
| **SARS** | Seryl-tRNA synthetase |
| **TARS** | Threonyl-tRNA synthetase |
| **VARS** | Valyl-tRNA synthetase |
| **WARS** | Tryptophanyl-tRNA synthetase |
| **YARS** | Tyrosyl-tRNA synthetase |

**Table S2.** TCGA cancer types and sample numbers per TCGA dataset. N: Normal, T: Tumour.

| Symbol | Name | DNA (N) | DNA (T) | mRNA (N) | mRNA (T) | tRNA (N) | tRNA (T) |
|---|---|---|---|---|---|---|---|
| BRCA | Breast Invasive Carcinoma | 139 | 1101 | 113 | 1092 | 104 | 1077 |
| HNSC | Head And Neck Squamous Cell Carcinoma | 74 | 528 | 44 | 518 | 44 | 523 |
| KIRC | Kidney Renal Clear Cell Carcinoma | 407 | 538 | 129 | 530 | 71 | 516 |
| KIRP | Kidney Renal Papillary Cell Carcinoma | 60 | 292 | 129 | 288 | 34 | 290 |
| LIHC | Liver Hepatocellular Carcinoma | 59 | 377 | 50 | 369 | 50 | 372 |
| LUAD | Lung Adenocarcinoma | 120 | 520 | 109 | 513 | 45 | 514 |
| LUSC | Lung Squamous Cell Carcinoma | 120 | 506 | 109 | 498 | 45 | 476 |
| PRAD | Prostate Adenocarcinoma | 67 | 498 | 52 | 495 | 52 | 483 |
| STAD | Stomach Adenocarcinoma | 102 | 478 | 36 | 414 | 37 | 409 |
| THCA | Thyroid Carcinoma | 65 | 507 | 59 | 504 | 71 | 510 |
